# Editorial: Challenges of Interdisciplinary Research in the Field of Critical (Sex/Gender) Neuroscience

**DOI:** 10.3389/fsoc.2021.797089

**Published:** 2022-01-12

**Authors:** Hannah Fitsch, Flora Lysen, Suparna Choudhury

**Affiliations:** ^1^ Zentrum für Interdisziplinäre Frauen- und Geschlechterforschung, Technical University of Berlin, Berlin, Germany; ^2^ Department Society Studies, Maastricht University, Maastricht, Netherlands; ^3^ Division of Social and Transcultural Psychiatry Montreal, McGill University, Montreal, QC, Canada

**Keywords:** sex/gender neuroscience, interdisciplinarity, intersectionality, feminist STS, neurogenderings, critical neuroscience, epistemic justice

There is currently widespread agreement among scholars that neuroscientific investigations that purport to delineate sex- and gender-related structural and functional brain differences urgently require conceptual critique, methodological nuance and thorough reflexivity about the research questions, operationalization, interpretations and implications shaping this scholarship (Fausto-Sterling, 2000; Fine, 2010; Jordan Young, 2010; Roy, 2012). In response to this need, the seven articles in this collection demonstrate new avenues in critical interdisciplinary scholarship in the field of sex/gender and neuroscience research, including approaches that draw on feminist science studies and critical neuroscience. Since the first publications that show how social and cultural values pervade the formulation of biological research on sex and gender, enormous developments have also occurred in the neurosciences, with increased evidence from functional neuroimaging and epigenetics pointing to the context-sensitivity and contingencies of brain development and function. This underscores the imperative for researchers to consider carefully their treatment of difference and of their conceptions of complexity and diversity. It is clear that we need to work out how to collaborate across epistemic boundaries, how to refine and draw on social theory to make sense of brain findings and how together this can inform interpretation of experimental data, data that bear relevance to the real world.

This Frontiers research topic builds on a key insight by critical feminist scholars: to arrive at a critical and more socially just production of knowledge about human behaviour it is important to go beyond the split between second order and first order observations, i.e., between critical sociological observations about neuroscientific practice and experimental investigations of the brain. Investigating and responding (to) this goal, the studies in this collection show how, in varying ways, scientific disciplines newly interact and may also clash in the formation of new conceptualizations of the relation between gender, sex and the material brain. The collection thus contributes to a better understanding of inter- or multi-disciplinary relations necessary to advance a study of the brain and human behaviour that is crucially informed by a feminist agenda. Moreover, improving our knowledge of (inter-)disciplinary epistemic dynamics by means of the specific case studies in this collection also offers background to an ongoing discussion about how to realize intersectional research.

The past 2 decades saw the emergence of a number of sub-(inter-)disciplinary labels and scholarly networks such as “critical neuroscience” (Choudhury and Jan, 2012, Kirmayer and Crafa, 2014), “neurofeminism” (Roy, 2008; Bluhm et al., 2012; Schmitz and Höppner, 2014), “neurogenderings” (Dussauge and Kaiser, 2012; Fitsch, 2012) and “neurocultures” (Schmitz and Höppner, 2014; Vidal and Ortega, 2018), as examples of the heterogeneous bodies of knowledge and gatherings of scholarship (sometimes converging, sometimes conflicting) that aim to analyse fundamental assumptions and particular biases in, as well as social, political and cultural contexts of, neuroscientific research and to address how studies of the body and the brain shape narratives about human behaviour, including gender difference (Kraus, 2012; Roy, 2012, 2016; Rippon et al., 2014; Kuria, 2014; O Connor and Helene, 2014; Joel and Fausto-Sterling, 2016; Bentley et al., 2019; Lockhart, 2020).

The current collection of articles builds on 2 decades of experimenting with forms of disciplinary collaboration in the examination of sex/gender and the brain (Fausto-Sterling, 2000) and takes important cues from scholars who have critically interrogated and problematized the hopes and forms of engagement attached to the buzzword “interdisciplinarity” (Callard and Fitzgerald, 2015). The question of interdisciplinarity–if and how scholars in the social sciences, humanities and biosciences should interact, inform, scrutinize or collaborate (with) one another to accomplish more nuanced and just articulations of biosociality–has been a longstanding and integral issue for (feminist) science and technology studies scholars, and a central challenge for critical neuroscientists, neurofeminists and other feminist (neuro-) science studies scholars of the brain and human cognition. In the past decade, feminist scholars have proposed new tools, models and experimental designs to generate more refined and socially just bio-socio-cultural perspectives in contemporary neuroscience. These researchers hotly contest sex/gender binaries in brain science (Joel, 2011; Schmitz and Höppner, 2014; Joel and Fausto-Sterling, 2016; Cornel, 2019; Walsh and Einstein, 2020; Eliot et al., 2021) and scrutinize the technological and statistical tools used in mapping sex/gender differences (Bryant et al., 2019; Sanchis-Segura et al., 2020; Duchesne et al., 2020; Eliot et al., 2021; Fitsch et al., 2020).

In a moment in which debates around the role of biology in relation to sex and gender is especially fraught, and indeed a moment in which the climate of debate within and beyond academia are particularly polarized, this conversation warrants particular reflexivity. Our goal in this issue is to invite views on what kinds of creative investigation may be most appropriate to address the question and unsettle existing assumptions (Fine, 2010), and the methodological challenges and potential they give rise to. Feminist neuroscientists have asked new, not purely binary, questions to data (Joel, 2011; Kaiser, 2012; Shattuck-Heidorn and Richardson, 2019; Eliot, 2020) and have come up with new models, such as the mosaic brain (Joel et al., 2015). Along with the critical examination of the apparatus of neuroscience, another important intervention into current practices of neuroscience is the work of feminist, queer and critical race studies scholars that raise issues of epistemic justice–of excluded bodies of knowledges and marginalized subjects, and rally for a “science from below” (Harding, 2008). A call for scholarship that works with people affected by the outcome, rather than studies that are about subjects, is prominent in disability studies, mental health and intersex/trans studies, in which the framework of epistemic justice has renewed the debate over critical studies of the normal and the pathological (Annamma et al., 2013; Baril, 2015; LeBlanc and Kinsella, 2016; Tremain, 2017). A third important development has come from scholars in (or partly affiliated to) critical race studies, who have generated renewed attention to colonial practices of (mis)measurements, surveying and administration of the marginalized (Heinz et al., 2014; Abiodun, 2019; Black in Neuro, 2021; Rollins, 2021b; Moody, 2021); have called for a decolonization of classificatory systems in neuroscience (Birhane and Guest, 2020); and emphasize the importance of developing a critical, intersectional perspective in accounts of humans in their environment (Alexander-Floyd, 2012; Collins and Bilge, 2020; Cole, 2020; Shields, 2008), including the study of sex/gender and the brain and relatedly, a critical perspective on institutional practices in neuroscience, including citation practices and grantee demographics (Choudhury and Neil, 2020; Dworkin et al., 2020). However, in spite of these significant sociologically-informed theoretical and methodological recommendations by feminist, queer and critical race studies scholars, such proposals are still under-used or haphazardly implemented in studies of the neuroscience of sex and gender.

Different figures or frameworks of disciplinary relationships have been in circulation: for example, the possibility of a more “critical friendship” between the social sciences/humanities and the life sciences to advance a non-reductionist articulation of human beings and other organisms in their milieu (Rose, 2013); or the call for a “dissensus studies” into sex/gender neuroscience, by which social scientists do not sidestep scientific controversy but exacerbate political matters by paying particular attention to social conflicts in relation to brain research (Kraus, 2016).

In the spirit of a call for “a more expansive account of human development and subject formation” (Frost, 2017), the papers in this collection demonstrate and critically analyse novel interdisciplinary relations to advance feminist and critical neuroscientific scholarship, examining fields ranging from fMRI research, brain-computer-interfaces and cyborgization, intersectionality in feminist psychology, infant gender/sex identity development, to brain studies of (trans)gender identity, neuro-epigenetics and trauma, and understandings of translational neuroscience literature on epigenetics.

## Seven Analyses of Interdisciplinarity in the Study of Sex/Gender and the Brain

20 years ago, Anne Fausto Sterling, contributor to the present collection of articles, predicted that cognitive scientists would have absorbed the important scholarship of feminist neuroscience into their research programs. “We will no longer be debating about male *versus* female brains or arguing that men are better than women at reading maps (…).” Writing in her seminal 2000-study *Sexing the Body*, she argued the way forward would be to create “non-hierarchical, multidisciplinary teams” to create awareness of the inevitable limits of disciplinary knowledge (Fausto-Sterling, 2000). Today, after two decades of path-breaking feminist advances in sex/gender research in the neuro- and life-sciences, it is clear that there is still much work to be done (Rippon et al., 2014; Bryant et al., 2019; Eliot et al., 2021).

In her contribution to this *Frontiers* collection of articles, Anne Fausto Sterling continues to emphasize the importance of designing interdisciplinary consortia that offer a meeting ground for insights from gender studies, neuroscience, physiology, developmental psychology and cognitive development. Based on extensive data analysis and literature review, her study proposes a multi-level, dynamic, and developmental systems theory of early gender/sex identity development and she discusses the challenges of understanding how infants integrate events that occur on different time scales and at different levels of biological integration. This theoretically-informed multi-level project can only be advanced, Fausto-Sterling argues, if researchers develop skills in interdisciplinary conversations and when they shape an emergent (not an additive) form of collaboration. Researchers need “to figure out how to draw conclusions that translate across levels of organismic organization (and disciplinary boundaries)”.

In their contribution for this collection, Lawson-Boyd and Meloni point to the need for more cross-disciplinary dialog in order to advance new perspectives on neuro-epigenetics. After an analysis of literature in the converging fields of neuro-epigenetics, sex/gender and trauma (with a particular focus on the work of feminist STS scholars), the authors evaluate a number of qualitative interviews they conducted with neuroscience and biology researchers in epigenetics and reflect on their interviewee’ knowledge of-and engagement with problems raised by feminist STS scholars. Lawson-Boyd and Meloni conclude that while scientists working in neuro-epigenetics have themselves raised the need for a reorientation of the field, they still have to take (more) knowledge from beyond the biosciences into account. If the aim (in the case of this field of scholarship) is to better understand and to ultimately reduce stress levels in mothers, a vital step, the authors argue, is a parallel analysis of “difference (and sameness) on the scales of neurophysiology and sociality.” This can only be done when researchers are willing to experiment with novel methodologies and when neuroscientists, molecular biologists and social scientists “speak candidly and respectfully with one another.”

The article by Norrmén-Smith et al. in this collection casts another perspective on the field of epigenetics, examining the impact of neurobiological and epigenetic framings of motherhood on pregnant women and new mothers. Based on detailed analysis of focus group data, they argue that the engagement of women with biomedical and cultural perinatal information on the internet and social media–for example, the discussion of the imprinting of mothers’ experiences on their prenatal baby’s DNA–has the potential to exacerbate emotional distress and to impact women’s experience, self-construal and wellbeing. The authors’ approach in this article is to bring a critical neuroscience-informed discourse analysis of neuroscience literatures around maternal and infant health together with qualitative analysis of focus group data about how consumers make sense of epigenetic and neuroscientific information and its looping effects. By taking this dual approach, the authors are careful not to overstate the transformative potential of popular neuroscientific rhetoric around plasticity and risk, but to study more closely how such information about brain-based susceptibility is interpreted and affects mothers. They demonstrate that while the appeal of neuroscience is often its state-of-the-art objectivity and novelty, it often ends up reinscribing the same social and moral dilemmas of older discourses, responsibilizing mothers in particular ways.

The articles by Schmitz and Fitsch in this collection emphasize the heterogeneity and interdisciplinary dynamics that are integral to the discipline of neuroscience itself. With a feminist STS-oriented discursive analysis, Schmitz examines current visions of transhumanism and the way these normative, discriminatory imaginaries of (the governing of) life are shaped and authorized by a body of neuroscientific research into brain-computer interfaces as well as discourses on neuro-technical developments. Paying attention to moments of inconsistency and recalcitrance in these systems, she proposes an alternative, more socially just articulation of “cyborgization.” The concept of cyborgization is meant to tackle the white, middle class, male rhetoric of grandiosity and modern neurobiological determinism and “the effects of neuro-technological and transhumanist governmentality on the question of whose lives are to be improved and whose lives should be excluded from these developments.”

Fitsch examines binary sex/gender categorization in magnetic resonance tomography and discusses empirical methodologies and epistemic underpinnings of differentiation through statistics. She argues that “counter-counting”, weighing and sizing is not helpful to substantiate the idea of “equality” (not only for sex/gender) in brain studies. The author asks for situated interdisciplinarity as “a scaffold” for intersectionality, to get epistemes, techniques and new methods on categorizing and differentiating in brain modelling into view. Referring to the topic of this special issue, this paper argues that for an interdisciplinary approach to criticize dimorphism and differentiation by groups, we need a broader understanding of the technical and theoretical foundations used in brain research.

Llaveria Caselles article for this collection points to the lack of interdisciplinary practices for advancing the study of (trans)gender identity. Llaveria Caselles employs the framework of epistemic injustice to analyse literature on brain studies of (trans)gender identity and to conduct an ethnomethodological study into the epistemic behaviours and attitudes of researchers involved in this field. In his article, Llaveria Caselles operationalizes “epistemic friction” by asking researchers about alternative, counter-hegemonic approaches to the study of (trans)gender. Llaveria Caselles identifies a lack of sensitivity towards biosocial, developmental, mosaicist, contextualist, and depathologizing research avenues and demonstrates the exclusion of counter-hegemonic practices and of epistemic agents associated with alternative approaches. He alludes to the way that systemic factors related to the organization of scientific work (such as the projectification of science) contribute to the privileging of “normal science” over revolutionary or risky science. To work towards a better and more just study of transgender identity, he recommends a number of strategic epistemic practices, including the “promotion of exchange across disciplines” and building inter- and transdisciplinary networks.

Llaveria Caselles’ study also points to the value of ethnomethodological research into the epistemic practices that foster or hinder intersectional approaches. Taking intersectionality seriously in the field of neuroscience means that researchers have to develop comprehensive analyses that include the tangled impacts and lived experiences of, for example, disability, race, sexuality, age, and class. Llaveria Caselles’ interviews with brain researchers demonstrate the problems of attending to intersectionality in experimental practice. One researcher pointed to the infeasibility of analysing how race, gender identity and context of upbringing interact with each other and affect brain development. Other reactions demonstrated misunderstanding of the concept of intersectionality all together. Overall, Llaveria Caselles concludes that researchers experienced “difficulties in moving away from a paradigm of clear categories, as well as the tendency to focus on biological and quantifiable factors.”

The issue of intersectionality is central to the final contribution to this collection, in which Duchesne and Kaiser Truijillo analyse how neurofeminist scholars may learn from intersectional approaches in feminist psychology scholarship. They point to three potentially valuable “research programs” in intersectional research in psychology literature and assess their value for feminist neuroscience. Duchesne and Kaiser Truijillo also address the problem of the gradual de-politicization and neutralization of (some versions of) intersectional research (away from the social justice-oriented change) and the move away from addressing the specific intersectional position of Black women. One potential means of addressing these issues in relation to the study of intersectionality, the authors argue, is to articulate the positionality of the authors and author’s scholarly relation to the intersectional objective of social justice. Again, understanding dynamics of disciplinary relations can help to advance an intersectional, feminist study of sex/gender and the brain.

## Conclusion

The articles in this collection provide the grounding for critical reflection on interdisciplinary approaches to sex/gender and the brain through various analytical examples from a range of scholarly backgrounds. Another outcome of this collection is that a number of contributions address–as part of a consideration of advancing novel forms and methodologies-the possibility and difficulties in conceptualizing and practicing intersectional approaches to the study of sex/gender and the brain. A closer look at inter-disciplinary, multi-disciplinary and trans-disciplinary research supports a more nuanced framework for the way ideas and methods can be drawn together to support such an intersectional approach. Key, in this respect, as various authors in this collection have mentioned, is attending to the interplay of various kinds of positionalities and embodiments to do justice to the plurality and complexity of human experience and to question practices of categorization.

In this vein, sociologist Oliver Rollins has recently argued that to gain a better understanding of under-examined practices of racialization in neuroscience, it is necessary to connect macro- and micro-level practices and to bring neuroscientific scholarship in conversation with social policy scholarship and to attend to the way neurobiological calculations may erroneously omit racial experiences or instead inadvertently encode normative ideas about racial worth (Rollins, 2021a; 2021b).

An interdisciplinary approach not only needs to open for other disciplinary perspectives, but also for new practices. Llaveria Caselles, in this collection, aligns his scholarly work with counter-hegemonic positions and calls for “interventionist projects” in knowledge production. Similarly, Lawson-Boyd and Meloni “urge scientists to consider what allowances and restrictions any positioned perspective offers.” Again, demands for other ways of doing science, are not new (Rose and Rose, 1979; Haraway, 1988). Some of these former calls should be reinvestigated to invigorate current approaches, to arrive at intersectional neuroscience and to improve our understanding of the interplay between science and society.
